# Phospho-mimetic CD3ε variants prevent TCR and CAR signaling

**DOI:** 10.3389/fimmu.2024.1392933

**Published:** 2024-05-08

**Authors:** Nadine M. Woessner, Simon M. Brandl, Sara Hartmann, Wolfgang W. Schamel, Frederike A. Hartl, Susana Minguet

**Affiliations:** ^1^ Faculty of Biology, University of Freiburg, Freiburg, Germany; ^2^ Signalling Research Centres BIOSS and CIBSS, University of Freiburg, Freiburg, Germany; ^3^ Spemann Graduate School of Biology and Medicine (SGBM), University of Freiburg, Freiburg, Germany; ^4^ Center of Chronic Immunodeficiency (CCI), University Clinics and Medical Faculty, University, Freiburg, Germany

**Keywords:** TCR - T cell receptor, CAR (chimeric antigen receptor), phospho-mimetic, LCK, CD3epsilon

## Abstract

**Introduction:**

Antigen binding to the T cell antigen receptor (TCR) leads to the phosphorylation of the immunoreceptor tyrosine-based activation motifs (ITAMs) of the CD3 complex, and thereby to T cell activation. The CD3ε subunit plays a unique role in TCR activation by recruiting the kinase LCK and the adaptor protein NCK prior to ITAM phosphorylation. Here, we aimed to investigate how phosphorylation of the individual CD3ε ITAM tyrosines impacts the CD3ε signalosome.

**Methods:**

We mimicked irreversible tyrosine phosphorylation by substituting glutamic acid for the tyrosine residues in the CD3ε ITAM.

**Results:**

Integrating CD3ε phospho-mimetic variants into the complete TCR-CD3 complex resulted in reduced TCR signal transduction, which was partially compensated by the involvement of the other TCR-CD3 ITAMs. By using novel CD3ε phospho-mimetic Chimeric Antigen Receptor (CAR) variants, we avoided any compensatory effects of other ITAMs in the TCR-CD3 complex. We demonstrated that irreversible CD3ε phosphorylation prevented signal transduction upon CAR engagement. Mechanistically, we demonstrated that glutamic acid substitution at the N-terminal tyrosine residue of the CD3ε ITAM (Y39E) significantly reduces NCK binding to the TCR. In contrast, mutation at the C-terminal tyrosine of the CD3ε ITAM (Y50E) abolished LCK recruitment to the TCR, while increasing NCK binding. Double mutation at the C- and N-terminal tyrosines (Y39/50E) allowed ZAP70 to bind, but reduced the interaction with LCK and NCK.

**Conclusions:**

The data demonstrate that the dynamic phosphorylation of the CD3ε ITAM tyrosines is essential for CD3ε to orchestrate optimal TCR and CAR signaling and highlights the key role of CD3ε signalosome to tune signal transduction.

## Introduction

The T cell antigen receptor (TCR) is a multimeric protein complex composed of the antigen-binding TCRαβ heterodimer and the non-covalently associated CD3 proteins, including the heterodimers CD3δε and CD3γε, and the ζζ homodimer (1, 2). Each CD3 subunit contains Immunoreceptor Tyrosine-based Activation Motifs (ITAMs) that mediate signal transduction. The cytoplasmic tails of CD3ε, CD3δ, and CD3γ each contain one ITAM, whereas ζ contains three ITAMs (3). Among the CD3 subunits, CD3ε is unique, since in addition to the ITAM, it contains other protein-protein interaction motifs in its cytoplasmic tail. The N-terminally located Basic Rich Sequence (BRS) recruits the Src family kinase LCK via LCK’s unique domain (4). Additionally, CD3ε interacts with the inner leaflet of the plasma membrane through the BRS (5). The Proline Rich Sequence (PRS) recruits the non-catalytic region of tyrosine kinase adaptor protein (NCK) via NCK’s first Src-homology 3 (SH3.1) domain and stabilizes the interaction of LCK with the TCR (6, 7). The recently discovered Receptor Kinase (RK) motif binds LCK via its SH3 domain (8). LCK and NCK form part of the signalosome of the un-phosphorylated CD3ε, but only when the TCR is in its active conformation, stabilized by antigen binding (6, 8, 9).

Although the structure of the ecto- and transmembrane-parts of the complete, resting TCR was resolved, a structure of all the cytoplasmic tails together remains an enigma (10). Biochemical experiments suggest that the TCR, including the cytoplasmic tails, switches between various conformational states (6, 11–13). Upon TCR engagement by agonist peptides bound to major histocompatibility complex (pMHC), the active TCR conformation is stabilized. This conformational change leads to the exposure of the ITAM, the PRS and the RK motif of CD3ε (6, 8, 9). Thereby, LCK is recruited via its SH3 domain to the RK motif of CD3ε, placing LCK in close proximity to the TCR allowing phosphorylation (8).

Dual phosphorylation of the ITAMs leads to the recruitment of the ζ-chain-associated protein kinase 70 (ZAP70) (14–16). A binding hierarchy of the tandem SH2 domain of ZAP70 to the ITAMs of ζ and CD3ε has been reported. The affinity is highest to the membrane proximal ITAM of ζ, followed by the second ITAM of ζ, and finally by the ITAM of CD3ε and the most distal ITAM of ζ (16, 17). Once bound to the TCR, ZAP70 becomes phosphorylated and activated. As a result, ZAP70, along with LCK, phosphorylates downstream signaling molecules to induce TCR-controlled signaling cascades resulting in T cell activation (18).

Since NCK and LCK recruitment to the antigen-bound TCR precedes ITAM phosphorylation, we aimed here to systematically investigate how the phosphorylation of each of the individual tyrosines of the CD3ε ITAM impacts the interaction with NCK, LCK and ZAP70. We mimicked tyrosine phosphorylation by mutating the tyrosines of the CD3ε ITAM to the negatively charged glutamic acid. The suitability of this approach was confirmed by taking advantage of the well-described phosphorylation-sensitive binding patterns of ZAP70 and NCK to CD3ε. In a step-wise approach, we demonstrated that exchanging each of the CD3ε ITAM tyrosines to glutamic acid mimics irreversible phosphorylation and prevents LCK binding. We demonstrate, that in the context of the TCR complex, dynamic CD3ε ITAM phosphorylation is required for optimal TCR signaling and ZAP70 phosphorylation, and that CD3ε phospho-mimetic variants of Chimeric Antigen Receptors (CARs) prevent CAR-mediated signal transduction.

## Materials and methods

### Cell lines

Human αβ Jurkat T cells (JK) (TIB-152, from ATCC), JK CD3ε CRISPR/Cas9 knock out cells (JK εKO, from TCR2 therapeutics), HEK293T cells (CRL-1573, from ATCC), CD19-expressing tumor B cells Nalm6 (from TCR2 therapeutics) and JK NFκB ϵKO and JK NFAT ϵKO reporter cell lines expressing GFP under the control of the indicated transcription factor were used in this study. The JK NFκB ϵKO and JK NFAT εKO were obtained by CRISPR/Cas9 technology, using the guide RNAs HS.Cas9.CD3E.1.AA: mG*mA*mU rGrUrC rCrArC rUrArU rGrArC rArArU rUrG and HS.Cas9.CD3E.1.AC: mA*mG*mG rGrCrA rUrGrU rCrArA rUrArU rUrArC rUrG (Integrated DNA Technologies).

JK εKO cells were transduced with lentivirus harboring the plasmids pLVX-SFFV-hCD3ε(WT)-FLAG-IRES-ZsGreen, pLVX-SFFV-hCD3ε(Y39E)-FLAG-IRES-ZsGreen, pLVX-SFFV-hCD3ε(Y50E)-FLAG-IRES-ZsGreen, pLVX-SFFV-hCD3ε(Y39/50E)-FLAG-IRES-ZsGreen to generate JK εKO human (h)CD3ε WT, Y39E, Y50E and Y39/50E, respectively. In addition, we generated JK NFκB and JK NFAT cell lines expressing the hCD3ε variants by transducing the different εKO reporter cell lines with lentivirus harboring the plasmids p526-mock-BFP, p526-hCD3ε(WT)-FLAG-T2A-BFP, p526-hCD3ε(Y39E)-FLAG-T2A-BFP, p526-hCD3ε(Y50E)-FLAG-T2A-BFP or p526-hCD3ε(Y39/50E)-FLAG-T2A-BFP.

To generate JK 19BBε CAR T cells, JK cells were transduced with lentivirus harboring the plasmids p526-19BBε(WT)-STREPII-T2A-GFP, p526-19BBε(Y39E)-STREPII-T2A-GFP, p526-19BBε(Y50E)-STREPII-T2A-GFP or p526-19BBε(Y39/50E)-STREPII-T2A-GFP. In addition, we generated JK NFκB CAR and JK NFAT CAR cell lines by transducing the εKO reporter cell lines with lentivirus harboring the plasmids p526-mock-BFP, p526-19BBε(WT)-FLAG-T2A-BFP, p526-19BBε(Y39E)-FLAG-T2A-BFP, p526-19BBε(Y50E)-FLAG-T2A-BFP or p526-19BBε(Y39/50E)-FLAG-T2A-BFP.

hCD3ε Y39E was generated by a single point mutation of the N-terminal CD3ε ITAM tyrosine (Y) 39 to glutamic acid (E) using primers CTTTCCGGATGGGCTCCTCGTCTGGGTTGGGAACA and TGTTCCCAACCCAGACGAGGA GCCCATCCGGAAAG. hCD3ε Y50E was generated by a single point mutation of Y50 to E using primers CTGATTCAGGCCAGACTCCAGGTCCCGCTGGCC and GGCCAGCGGGACCTGGAGTCTGGCCTGAATCAG. hCD3ε Y39/50E was generated by introducing the Y50E mutation into the hCD3ε Y39E construct. The sequencing primer CGGGGCAGAAAGAAACTG was used for all 19BBε constructs and the sequencing primer GACCTCCATAGAAGACACC was used for the hCD3ε constructs.

DMEM (Gibco) enriched with 10% heat-inactivated fetal bovine serum (FBS), 10 mM HEPES (Gibco), and 50 U/ml penicillin-streptomycin (Gibco) was used to culture HEK293T cells at 37°C in a humidified incubator with 7.5% CO_2_. All JK-derived cell lines were cultured in RPMI 1640 media (Gibco) supplemented with 10% FBS, 10 mM HEPES, 50 U/ml penicillin-streptomycin at 37°C with 5% CO_2_.

### Antibodies

Antibodies used for western blot development: anti-NCK1 (15B9, Cell Signaling), anti-ZAP70 (1E7.2, Santa Cruz Biotechnology or Cell Signaling), anti-LCK (3A5, Santa Cruz Biotechnology), anti-CD3ε (Everest Biotechnology), anti-ζ antiserum 449, anti-GST (Bethly), anti-GAPDH (Sigma), anti-pZAP70 (Y319) (Cell Signaling), anti-pLCK (Y505) (Cell Signaling), anti-pLCK (Y394) (Cell Signaling) and secondary antibodies for immunoblotting (Perbio). Antibodies used for cell stimulation: anti-CD3ε (UCHT1 or OKT3); anti-CD28.2 (BioLegend). Antibodies used for flow cytometry: Alexa Fluor 647- or Brilliant violet-421-labeled anti-CD3ε (UCHT1, BioLegend), PE-conjugated anti-CD3 (OKT3, eBioscience), PE-conjugated anti-CD28 (Biolegend). Either APC-labeled anti-Flag Tag antibody (BioLegend), anti-Strep Tag II antibody (Genscript) or anti-mouse IgG F(ab’)2 (Invitrogen) followed by streptavidin-APC or streptavidin-FITC (BioLegend) were used for the detection of surface CARs. In addition, PE-Cy7-labeled anti-CD137, PE-labeled anti-CD107a, and PE-labeled anti-CD25 all from BioLegend; APC-labeled anti-CD69 (Life Technologies) were used.

### Production of lentiviruses

10^7^ HEK293T cells were transfected using PEI transfection with the appropriate constructs and the packaging plasmids pCMVR8.74 (gag/pol) and pMD2.G (envelope). The virus-containing supernatant was collected 24 h and 48 h after transfection. Virus-containing supernatant was concentrated for 4 h at 10.000 xg and 6°C using a 10% sucrose gradient (supplemented with 0.5 mM EDTA). The concentrated virus was resuspended in 100 μl 0% FBS RPMI and frozen at -80°C until use.

### Transduction of Jurkat T cells

2x10^5^ cells were transduced with a virus multiplicity of infection (MOI) of 4 for 72 h. Cells were then washed and transduction efficiency was assessed by flow cytometry 5-7 days after transduction.

### Primary human T cell activation, transduction, and expansion

Cryopreserved purified peripheral blood mononuclear cells (PBMCs) from healthy donors were thawed and resuspended in 10% FBS RPMI supplemented with 500 U/ml recombinant human IL-2 (PeproTech) and activated with plate-bound anti-CD3 (UCHT1, homemade) and anti-CD28.2 (BioLegend) (1 μg/ml). 48-72 h after activation, >99% of the cells were T cells. Activated primary human T cells were lentivirally transduced using spin infection with a virus MOI of 4 together with 5 μg/ml of protamine sulfate (Sigma) and 500 U/ml of recombinant human IL-2. 8-12 days after transduction, CAR surface levels were determined by flow cytometry. After transduction, cells were grown in 10% FBS RPMI supplemented with 100 U/ml IL-2 before being used for the indicated experiments.

### Cell stimulation and lysis

For flow cytometry analysis, 1.5-2x10^5^ cells were resuspended in 200 μl 10% FBS RPMI. Cells were either left unstimulated, stimulated with 5 μg/ml plate-bound anti-CD3ε antibody (UCHT1), stimulated with Nalm6 cells in an effector to target ratio (E:T) of 1:1, or stimulated with 10 ng/ml phorbol myristate acetate (PMA) and 250 ng/ml ionomycin. Surface levels of activation markers were assayed after 24 h of stimulation. For pull-down experiments 30x10^6^ JK cells per sample were lysed in 1 ml of EMBO lysis buffer containing 20 mM Tris-HCl (pH 8), 137 mM NaCl, 2 mM EDTA, 10% glycerol, 1 μg/ml protease inhibitor cocktail (Sigma), 1 mM PMSF, 5 mM iodoacetamide, 0.5 mM sodium orthovanadate, 1 mM NaF and 0.3% Brij96V for 1 h on ice. For immunoblotting, 2x10^6^ JK cells per sample were resuspended in 100 μl of 0% FBS RPMI and starved for 1 h at 37°C. Cells were then stimulated with 5 μg/ml anti-CD3ε antibody (UCHT1) for 5 min at 37°C. Cells were lysed in 100 μl of EMBO lysis buffer for 30 min on ice, followed by a 20 min centrifugation at 12.000 g, then the supernatant was stored at -20°C until use.

### Pull-down assay and Immunoblotting

For the PD assay, the different phospho-mimetic variants of cytoplasmic tails of human CD3ε were cloned into the pRP261 vector and fused C-terminally to GST. The *E.coli* strain BL21 was transformed with the corresponding vectors. After 3 h induction with 1 mM IPTG, bacteria were lysed. GST-CD3ε fusion proteins were purified using glutathione sepharose beads (GE Healthcare). CD3ε PD assay was performed overnight at 4°C. After washing, proteins were separated by SDS-PAGE and immunoblotted in a semi-dry chamber for 1 h at 18 V. Chemiluminescence was used to image protein bands using a CCD camera (ImageQuant LAS 4000; GE Healthcare). ImageJ and ImageQuantTL software were used to determine the relative band intensity (GE Healthcare).

### Enzyme-linked immunosorbent assay

2x10^5^ cells incubated in 200 μl 10% FBS RPMI were either left unstimulated or stimulated with 5 μg/ml plate-bound anti-CD3ε antibody (UCHT1) and 1 μg/ml soluble anti-CD28 antibody, stimulated with Nalm6 cells using a 1:1 ratio, or stimulated with 10 ng/ml PMA and 250 ng/ml ionomycin. After 24 h, the supernatants were collected and cytokine levels determined using commercial ELISA Kits (eBioscience).

### Intracellular calcium influx

5x10^5^ cells per sample were resuspended in 1% FBS RPMI and incubated for 30 min at 37°C with 5 μg/ml of Indo-1 and 0.5 μg/ml of pluronic F-127 (Molecular Probes). After washing, cells were kept in the dark on ice. Right before measurement, cells were pre-warmed 5 min at 37°C. The Ca^2+^ response was induced by addition of 0.5 μg/ml anti-CD3ε (UCHT1) after recording 60 s of baseline. The change of the ratio Indo-bound versus Indo-unbound was measured with a MaxQuantX Flow Cytometer (Miltenyi Biotech). Data were normalized to the baseline with FlowJo software version 9.3.2.

### *In situ* proximity ligation assay

1x10^5^ cells per sample were starved in 0% FBS RPMI and rested on diagnostic microscope slides (Thermo Fisher Scientific) for 1 h at 37°C. Cells were left unstimulated or stimulated with 5 μg/ml anti-CD3ε (OKT3) for 5 min at 37°C. Cells were then fixed in 2% PFA for 15 min, permeabilized with 0.5% saponin for 30 min and blocked. Blocked cells were stained according to the manufacturer’s instructions with the Duolink kit (Olink Bioscience) with goat anti-CD3ε (Everest Biotechnology) and mouse anti-LCK (3A5, Santa Cruz Biotechnology). Nuclei were stained with DAPI (Roth). A total of 5–7 images (on average 700 cells) per sample were taken at ×60 with a confocal microscope (Nikon C2) and analyzed with BlobFinder.

### Bioluminescence-based cytotoxicity assay

3x10^4^ primary CAR T cells were cultivated in 10% FBS RPMI supplemented with 75 μg/ml D-firefly luciferin potassium salt (Biosynth) and co-cultured with luciferase-expressing Nalm6 tumor cells using a 1:1 ratio. Bioluminescence (BLI) was measured in a luminometer (BioTek Synergy H4 Hybrid Reader) as relative light units (RLUs). Maximal cell death was defined by the RLU signals from cells treated with 1% Triton X-100 and unspecific tumor cell lysis was set by the RLU signals from target cells incubated with non-transduced T cells. Percent specific tumor cells lysis (specific killing) was calculated with the following formula: % specific tumor cell lysis = 100 × (average non-transduced T cell killing RLU − test RLU)/(average non-transduced T cell killing RLU − average maximal death RLU).

### Degranulation assays

2x10^5^ CAR T cells were cultured with Nalm6 cells using a 1:1 ratio in the presence of 1 μl of PE-labelled anti-CD107α antibody for 3 h. Cells were then stained with a PE-labelled anti-CD107α antibody and surface levels were assessed by flow cytometry.

### Quantification and statistical analysis

Figures and figure legends provide statistical parameters such as the actual value of n, precision measurements (mean ± SD), and statistical significance. Differences were considered significant when the p values were < 0.05. Data sets were tested for normality using the Shapiro-Wilk test. Statistical significance in each figure was calculated as indicated in the figure legend. Only statistically significant differences are shown. Statistical analysis was performed in GraphPad PRISM 10.

## Results

### LCK binding to recombinant CD3ϵ is abolished by mimicking irreversible phosphorylation of Y50E

**Figure 1:**
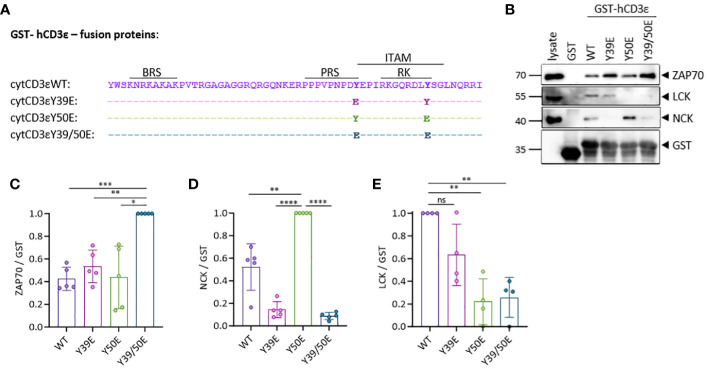
CD3ε phospho-mimetic variants bind distinct signaling molecules. **(A)** Sequence of the human CD3ε-fusion proteins used in the study. **(B)** Pull-down (PD) assay in Jurkat (JK) T cell lysates using beads coupled to the human cytoplasmic CD3ε tail fused to GST and followed by immunoblotting with the indicated antibodies. **(C–E)** Quantification of 4-5 independent experiments done as in **(B)**. ZAP70 **(C)**, NCK **(D)** and LCK **(E)** signals were normalized to GST for each sample. One sample t test was performed after Shapiro-Wilk test for normality. Mean values ± SD are indicated. Each dot represents one independent experiment. Ns, non-significant; *P < 0.5, **P < 0.01, ***P < 0.001, ****P < 0.0001.

It has been previously shown that LCK binds with its SH3 domain to the un-phosphorylated cytoplasmic tail of CD3ε (8). It was demonstrated using phosphorylated peptides that this binding was reduced when the CD3ε ITAM was doubly phosphorylated. Molecular modeling predicted that the OH group of the second tyrosine (Y50, numbered based on the sequence shown in **Figure 1A**) of the CD3ε ITAM forms a hydrogen bond with a serine from LCK (8). This interaction should be abolished upon Y50 phosphorylation, explaining why the SH3(LCK) domain failed to bind the phosphorylated cytoplasmic tail of CD3ε. We aimed here to experimentally confirm this hypothesis and to systematically assay the role of each CD3ε ITAM tyrosines for LCK binding.

To mimic phosphorylation, we mutated each CD3ε ITAM tyrosine (Y), individually or both simultaneously, to glutamic acid (E) (**Figure 1A**). We generated GST-fusion proteins containing the different phospho-mimetic variants of the cytoplasmic tail of CD3ε (WT, Y39E, Y50E, Y39/50E) and used them for pull-down assays with the lysate of Jurkat (JK) T cells (**Figure 1B**). Using phospho-peptides, it has been demonstrated that ZAP70 binds with its tandem SH2 domain to doubly phosphorylated ITAMs (8, 19). In our experiments, ZAP70 bound significantly better to the CD3ε Y39/50E variant than to WT CD3ε or the single mutants (**Figures 1B, C**). NCK recruitment to the TCR occurs via the interaction between the SH3.1 domain of NCK and the PRS of CD3ε (6), and phosphorylation of Y39 interrupts this interaction as demonstrated using phosphorylated peptides (20, 21). As expected, NCK bound to CD3ε WT and CD3ε Y50E, but not to the Y39E or Y39/50E variants in our experiments (**Figures 1B, D**). Binding to CD3ε Y50E was enhanced compared to CD3ε WT, being in line with data suggesting that the SH2 domain of NCK binds to phosphorylated Y50 (21). Hence, we show here that mutation of tyrosine to glutamic acid is able to mimic tyrosine phosphorylation of the CD3ε ITAM, confirmed by the specific binding pattern of ZAP70 and NCK. LCK binding to CD3ε was reduced by 80% for CD3ε Y50E and Y39/50E variants compared to CD3ε WT, confirming our previous hypothesis that phosphorylation of Y50 abolishes LCK binding to CD3ε (**Figures 1B, E**). Altogether, these results show that LCK binding to the cytoplasmic tail of CD3ε is phosphorylation-sensitive, suggesting that Y50 acts as a molecular switch regulating the interaction of the full-length LCK with CD3ε.

### Phospho-mimetic variants of the CD3ε ITAM tyrosines reduce T cell activation

**Figure 2:**
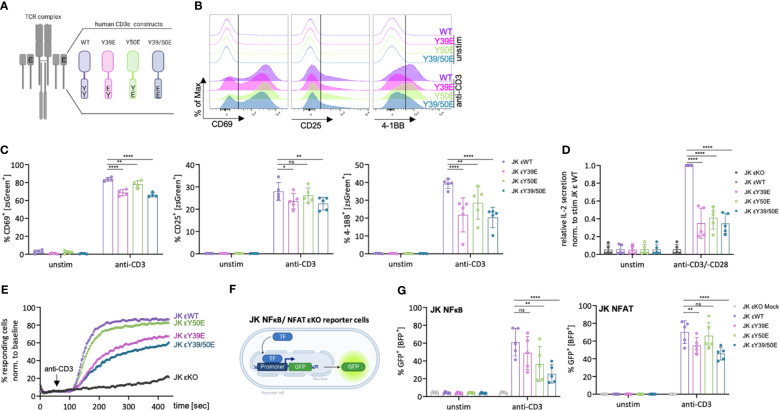
Reduced activation of JK T cells expressing phospho-mimetic CD3ε variants. **(A)** Schematic depiction of the human CD3ε (hCD3ε) variants used in this study. **(B, C)** JK εKO cells expressing the respective hCD3ε variants were left unstimulated or stimulated with 5 μg/ml anti-CD3 antibody for 24 (h) Representative histograms **(B)** and the percentage of CD69^+^, CD25^+^ or 4-1BB^+^ cells **(C)** was assessed by flow cytometry. 4-5 independent experiments are pooled. **(D)** JK εKO cells expressing the indicated hCD3ε variants were left unstimulated or stimulated with 5 μg/ml anti-CD3 and 1 μg/ml anti-CD28 antibody for 24 (h) The relative IL-2 secretion was assessed by ELISA. 5 independent experiments were performed and each normalized to the stimulated εWT sample. **(E)** Percentage of Ca^2+^ fluxing cells assessed by flow cytometry upon stimulation with 0.5 μg/ml anti-CD3 antibody. One representative experiment out of three independently performed experiments is shown. **(F)** JK εKO reporter cells, in which GFP expression is regulated by the responsive elements of NFκB, or NFAT, were transduced with a mock vector or the respective hCD3ε variants. **(G)** JK NFκB/NFAT cells were left unstimulated or stimulated with 5 μg/ml anti-CD3 antibody for 24 (h) Percentage of GFP^+^ cells was assessed by flow cytometry. 5 independent experiments are pooled. Two-way ANOVA with Dunnett’s multiple comparisons was performed after Shapiro-Wilk test for normality. Mean values ± SD are indicated. Each dot represents one independent experiment. Ns, non-significant, *P < 0.5, **P < 0.01, ****P < 0.0001.

Next, we aimed to investigate whether mimicking irreversible phosphorylation of the CD3ε ITAM tyrosines impacts signal transduction in the context of the complete TCR. To this end, we expressed four variants of CD3ε (WT, Y39E, Y50E, Y39/50E) in Jurkat (JK) T cells with a CRISPR/Cas9 knock-out for CD3ε (JK εKO) (**Figure 2A**). TCR and CD28 cell surface levels were equal in all generated cell lines (**Supplementary Figure 1**).

The phospho-mimetic CD3ε variants did not transduce activation signals in the absence of stimulus, demonstrated by the lack of CD69, CD25 and 4-1BB up-regulation, as well as IL-2 secretion under tonic conditions (**Figures 2B–D**). Upon stimulation with an anti-CD3 antibody for 24 hours, JK T cells expressing CD3ε WT up-regulated CD69, CD25 and 4-1BB (**Figures 2B, C**). The up-regulation of these activation markers was reduced by 10-40% in JK T cells expressing the phospho-mimetic CD3ε variants. IL-2 production in JK T cells expressing the phospho-mimetic CD3ε variants was strongly reduced (60%) when compared to their WT counterpart (**Figure 2D**). These results suggest that irreversible phosphorylation of the CD3ε ITAM could impact optimal T cell activation. However, it also suggests some redundancy between the ten ITAMs of the TCR-CD3 complex, because in none of the phospho-mimetic variants signaling was completely abolished. The multitude of ITAMs might contribute to signal amplification and/or diversity. Nevertheless, evidences support that some degree of redundancy between the CD3 ITAMs is tolerated during T cell development (22).

We next tested for intracellular Ca^2+^ mobilization upon TCR triggering. The CD3ε Y50E variant slightly, but consistently, reduced the percentage of Ca^2+^ responding cells (**Figure 2E**). This result is in line with our previous study showing that reducing LCK recruitment by mutating the RK motif, leads to a slight reduction of Ca^2+^ responding cells (8). Expression of the Y39E and the Y39/50E variants resulted in a stronger reduction of the percentage of responding cells when compared to the CD3ε WT (**Figure 2E**), suggesting that abolishing NCK recruitment, leads to a stronger reduction of Ca^2+^ responding cells as previously proposed by mutating the PRS motif (8).

Next, we tested whether the phospho-mimetic CD3ε variants are able to activate the T cell transcription factors NFκB and NFAT. Therefore, we used JK-derived reporter cells, in which GFP is expressed under the control of NFκB or NFAT (23) (**Figure 2F**), after performing a CRISPR/Cas9 knock out of CD3ε to prevent signaling through the endogenous TCR. The efficiency of the εKO was confirmed by flow cytometry and western blot analysis (**Supplementary Figures 2A–C**). Then we transduced the reporter cell lines with the respective CD3ε variants and confirmed restored TCR surface levels by flow cytometry (**Supplementary Figures 2D–M**). Activation for 24 h with an anti-CD3 antibody resulted in activation of NFκB in the JK NFκB CD3ε WT cells, indicated by the increase of GFP^+^ cells. Expression of the CD3ε Y50E and Y39/50E variants reduced this activation by up to 50% (**Figure 2G**). In the JK NFAT reporter cells, GFP expression was reduced upon expression of the CD3ε Y39E and Y39/50E variants compared to WT, while the CD3ε Y50E variant did not affect NFAT activation in line with our Ca^2+^ influx results (**Figures 2E, G**).

**Figure 3:**
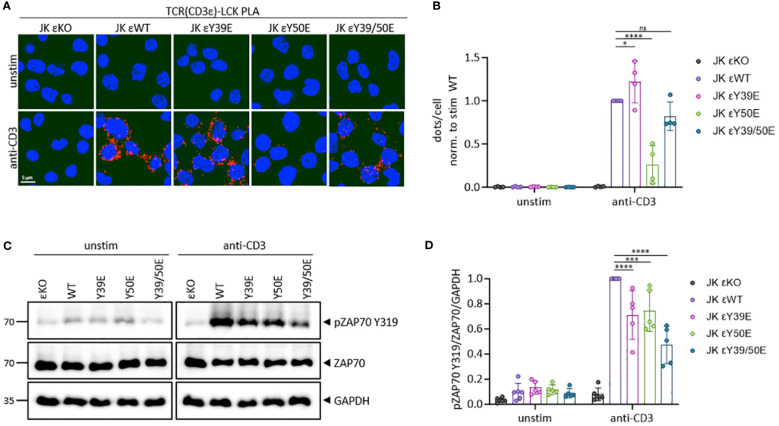
Phospho-mimetic variants of CD3ε define TCR-LCK proximity and diminish ZAP70 phosphorylation in JK cells. **(A)** JK εKO cells expressing the respective phospho-mimetic hCD3ε variant were either left unstimulated or stimulated with 5 µg/ml anti-CD3 antibody for 5 min at 37°C. A proximity ligation assay (PLA) was performed between the TCR (CD3ε) and LCK. A red dot indicates a proximity closer than 80 nm. Images from one representative experiment are shown. Cell nuclei are stained with DAPI. **(B)** Quantification of four independent PLA experiments. Dots per cell are normalized to the stimulated WT for each experiment. **(C)** Total cell lysates were subjected to immunoblotting with anti-pZAP70 (Y319), anti-ZAP70, and anti-GAPDH antibodies. JK εKO cells were used as negative control. **(D)** The quantification of 5 independent experiments normalized to the stimulated WT is shown. Two-way ANOVA with Dunnett’s multiple comparisons test was performed after Shapiro-Wilk test for normality. Mean values ± SD are indicated. Each dot represents one independent experiment. Ns, non-significant, *P < 0.5, ***P < 0.001, ****P < 0.0001.

To deeper investigate the mechanisms behind the reduced signal transduction of the phospho-mimetic CD3ε variants, we performed an *in situ* proximity ligation assay (PLA) between the TCR (CD3ε) and LCK. To this end, the JK εKO cells reconstituted with the CD3ε WT, Y39E, Y50E or Y39/50E construct were either left unstimulated or stimulated with an anti-CD3 antibody. Upon stimulation, the proximity of the TCR and LCK strongly increased in the CD3ε WT expressing cells (**Figures 3A, B**). The proximity between the TCR and LCK was slightly increased by the Y39E variant, while the Y50E variant reduced TCR-LCK proximity by 60%. The TCR-LCK proximity for the Y39/50E variant was reduced by 30% compared to the WT, which might be explained by binding of LCK to other phosphorylated ITAM motif, beyond CD3ε, with its SH2 domain (**Figures 3A, B**). This pattern was also observed in our previous study, where reduced NCK recruitment by a PRS depletion did not affect TCR-LCK proximity, while reducing LCK recruitment by mutating the RK motif, strongly reduced TCR-LCK proximity compared to CD3ε WT (8). LCK activity is regulated by the phosphorylation status of its regulatory tyrosines Y394 and Y505 (24). Phosphorylation of these tyrosine residues was unaffected by the phospho-mimetic CD3ε variants (**Supplementary Figure 3**).

We next studied the phosphorylation status of ZAP70. LCK phosphorylates ZAP70 at Y315 and Y319 to unlock the autoinhibited conformation of ZAP70. This stabilizes its binding to phosphorylated ITAMs and facilitates catalytic activity and downstream signaling to activate T cells (25). JK εKO cells expressing CD3ε WT or the phospho-mimetic CD3ε variants were left unstimulated or stimulated with an anti-CD3 antibody. Total cell lysates were analysed for ZAP70 phosphorylation at Y319. JK εKO cells were used as negative control. In JK εKO cells reconstituted with CD3ε WT, Y319 phosphorylation of ZAP70 increased upon TCR triggering (**Figures 3C, D**). In contrast, the Y319 phosphorylation of ZAP70 upon stimulation was significantly decreased by 30-50% in cells expressing the phospho-mimetic CD3ε variants (**Figures 3C, D**).

Altogether, mimicking the irreversible phosphorylation of the CD3ε ITAM reduces phosphorylation of ZAP70, and thereby prevents efficient downstream signaling and T cell activation. We suggest that each CD3ε phospho-variant prevents optimal TCR activation by different means. Y39E allows the binding of LCK, but the premature recruitment of CSK (26) might prevent the catalytic activity of LCK. Y50E can bind NCK but not LCK and therefore is unable to transduced effective signaling. Y39/50E can bind ZAP70, however, in the absence of LCK binding, ZAP70 might not be efficiently activated. Importantly, these data suggest a specific and non-redundant role for the CD3ε ITAM in signal initiation, but also highlights some degree of compensation by any of the additional ITAMs present in the TCR-CD3 complex.

### Phospho-mimetic variants of a CD3ε-containing chimeric antigen receptor impair T cell activation

**Figure 4:**
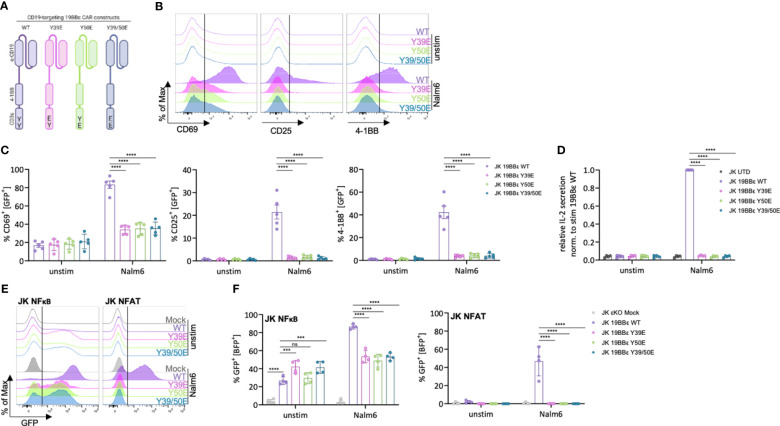
Reduced activation and signaling capacity of JK T cells expressing phospho-mimetic CAR variants. **(A)** Schematic depiction of the CD19-targeting CAR constructs (19BBε) used in this study. **(B–D)** JK cells expressing the indicate 19BBε constructs were left unstimulated or stimulated with CD19^+^ Nalm6 tumor cells (effector to target ration (E:T) 1:1) for 24 (h) Representative histograms **(B)** and the percentage of CD69^+^, CD25^+^ or 4-1BB^+^ cells **(C)** are shown from five independent experiments. The relative IL-2 secretion **(D)** is shown for four independent experiments each normalized to the stimulated WT sample. **(E, F)** JK NFκB/NFAT εKO reporter cells were transduced with a mock construct or the respective 19BBε variants. Cells were left unstimulated or stimulated with CD19^+^ Nalm6 tumor cells for 24 (h) Representative histograms **(E)** and the percentage of GFP^+^ cells **(F)** are shown. Four independent experiments were pooled. Two-way ANOVA with Dunnett’s multiple comparisons test was performed after Shapiro-Wilk test for normality. Mean values ± SD are indicated. Each dot represents one independent experiment. Ns, non-significant, ***P < 0.001, ****P < 0.0001.

We next designed minimalistic TCRs in the form of CD3ε-containing CARs to solely investigate the CD3ε ITAM and to avoid compensation by other ITAMs, as it is the case in the context of the TCR-CD3 complex. To this end, we engineered CAR constructs by exchanging the ζ to the CD3ε cytoplasmic tail in the FDA-approved second-generation CAR containing the co-stimulatory domain of 4-1BB (CD137) and targeting CD19. We have previously shown that a CD3ε containing CAR outperformed the FDA-approved ζ CARs in a preclinical model (27). In addition to CD3ε WT, we have here generated CARs containing the phospho-mimetic variants of CD3ε. These CARs will be named from now on 19BBε WT, 19BBε Y39E, 19BBε Y50E and 19BBε Y39/50E (**Figure 4A**). All constructs were expressed on the surface of JK T cells, which neither express CD4 nor CD8 (8), and all express CD28 similarly, as verified by flow cytometry (**Supplementary Figure 4**).

Strong tonic signaling is a major limitation of CARs that can lead to early CAR T cell exhaustion and to excessive cytokine secretion (28). We did not observe increased tonic signaling when the phospho-mimetic 19BBε variants were compared to the WT neither by CD69, CD25 or 4-1BB up-regulation, nor by IL-2 secretion (**Figures 4B–D**). Next, all JK 19BBε variants were co-incubated at a 1:1 ratio with the CD19^+^ B cell precursor leukaemia cell line Nalm6 for 24 hours. Cells expressing the 19BBε WT CAR were effectively activated, as shown by up-regulating CD69, CD25 and 4-1BB, as well as by IL-2 secretion (**Figures 4B–D**). In contrast, all phospho-mimetic CAR variants failed to transmit activation signals as seen by lack of up-regulation of CD69, CD25 and 4-1BB as well as IL-2 secretion above background (**Figures 4B–D**).

To deeper investigate the signaling defects observed by the phospho-mimetic 19BBε variants, we transduced the JK NFκB εKO and JK NFAT εKO reporter cell lines with a mock construct or the CAR variants. All 19BBε CARs were expressed on the cell surface of the reporter cell lines (**Supplementary Figure 5**). It is well known that the tumor necrosis factor receptor (TNFR) family, such as 4-1BB, activates the NFκB pathway (29). Indeed, all 19BBε variants activated NFκB as seen by the increase in GFP^+^ cells in the absence of ligand compared to the mock-expressing control cells (**Figures 4E, F**). In contrast, no tonic activation of NFAT was observed (**Figures 4E, F**). Upon co-incubation with CD19^+^ Nalm6 cells, 19BBε WT CARs significantly activated the transcription factors NFκB and NFAT (**Figures 4E, F**). In comparison with the 19BBε WT CAR, the three phospho-mimetic CAR variants (Y39E, Y50E, Y39/50E) failed to further increase 4-1BB-mediated NFκB activation (**Figures 4E, F**). Likewise, all phospho-mimetic 19BBε variants prevented activation of the transcription factor NFAT upon ligand binding to the CARs (**Figures 4E, F**). Taken together, these results indicate that mimicking constitutive tyrosine phosphorylation of the CD3ε ITAM prevents signal transduction by CARs and thus, T cell activation in JK cells.

### Phospho-mimetic variants of CD3ε prevent CAR activation in primary human T cells

**Figure 5:**
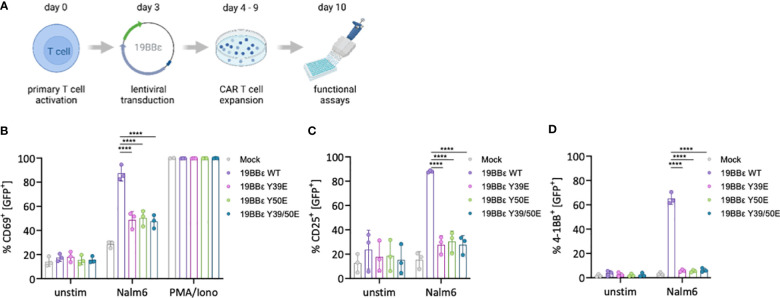
Phospho-mimetic CAR variants prevent T cell activation in primary human CAR T cells. **(A)** Schematics shows how PBMCs were activated with 1 μg/ml anti-CD3 and anti-CD28, and then lentivirally transduced to generate the respective 19BBε CAR T cells. **(B–D)** Primary CAR T cells were either left unstimulated, stimulated with CD19^+^ Nalm6 tumor cells (E:T 1:1), or with 10 ng/ml PMA and 250 ng/ml ionomycin for 24 (h) The percentage of CD69^+^ **(B)**, CD25^+^ **(C)** and 4-1BB (CD137)^+^ **(D)** cells were assessed by flow cytometry. Three different healthy donors were used. Two-way ANOVA with Dunnett’s multiple comparisons was performed after Shapiro-Wilk test for normality. Mean values ± SD are indicated. Each dot represents one healthy donor. ****P < 0.0001.

We next investigated the impact of the phospho-mimetic 19BBε variants in a more physiological and translational-orientated system by expressing them in primary human peripheral blood T cells (**Figure 5A**). All CARs were expressed at similar levels as assayed by flow cytometry (**Supplementary Figure 6**). Tonic signaling was comparable between the phospho-mimetic 19BBε variants and WT CAR as shown by neither CD69, CD25 nor 4-1BB up-regulation under basal conditions (**Figures 5B–D**). Triggering of the 19BBε WT CAR by co-incubation of primary CAR T cells with CD19^+^ Nalm6 cells efficiently induced T cell activation, with a significant increase in the proportion of cells expressing CD69, CD25 and 4-1BB (**Figures 5B–D**). In marked contrast, all phospho-mimetic 19BBε variants failed to optimally induce T cell activation (**Figures 5B–D**). Stimulation with PMA and ionomycin, bypassing the membrane proximal signaling events of the CAR, resulted in maximal CD69 up-regulation in all 19BBε CAR variants (**Figure 5B**). This control demonstrates that all CAR T cells have the same capacity to up-regulate CD69, excluding intrinsic differences in T cell differentiation induced by the individual CAR constructs. These results substantiate our hypothesis that irreversible phosphorylation of 19BBε CARs impairs signal transduction upon CAR triggering.

### Reduced and delayed cytotoxicity as well as cytokine secretion by primary CAR T cells expressing CD3ε phospho-mimetic CAR variants

**Figure 6:**
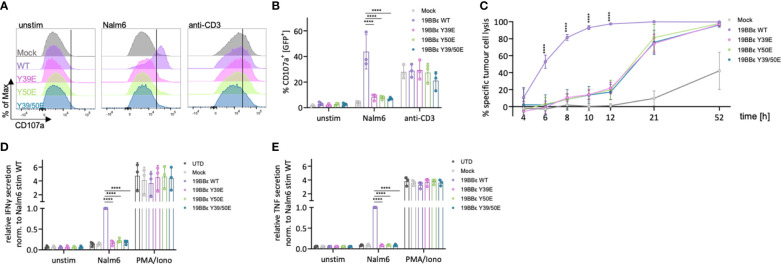
Reduced cytotoxicity and cytokine secretion of primary T cells expressing phospho-mimetic CAR variants. **(A, B)** Primary CAR T cells expressing the respective 19BBε CAR constructs were either left unstimulated, stimulated with CD19^+^ Nalm6 tumor cells (E:T 1:1) or stimulated with 5 μg/ml anti-CD3 antibody for 3 (h) Representative histograms for CD107a **(A)** and the percentage of CD107a^+^ cells **(B)** are shown. Two-way ANOVA was performed after Shapiro-Wilk test for normality. Each dot represents one healthy donor. **(C)** Time course showing the specific tumor cell lysis of 19BBε CAR T cell variants upon co-incubation with CD19^+^ Nalm6 tumor cells. **(D, E)** Primary CAR T cells were either left unstimulated, stimulated with CD19^+^ Nalm6 tumor cells (E:T 1:1) or stimulated with 10 ng/ml PMA and 250 ng/ml ionomycin for 24 h and IFNγ **(D)** as well as TNF **(E)** secretion was assessed by ELISA. Each experiment was normalized to the respective Nalm6-stimulated 19BBε WT sample. Two-way ANOVA with Dunnett’s multiple comparisons was performed after Shapiro-Wilk test for normality. 3 different healthy donors were used. Each dot represents one healthy donor. Mean values ± SD are indicated. ****P < 0.0001.

CAR T cells mediate their anti-tumoral effects through directly killing tumor cells, as well as by the release of cytokines to sensitize the tumor stroma. Thus, we investigated the cytotoxic activity of primary CAR T cells expressing our novel 19BBε CARs. CAR T cells mediate cytotoxicity mainly through the perforin and granzyme axis. Therefore, we first tested for the degranulation of primary CAR T cells upon encounter with tumor cells. This process results in the fusion of the granule membrane with the plasma membrane of the CAR T cell, causing the surface exposure of proteins that are present on the membrane of the lytic granules, such as CD107a. The protein CD107a can be detected using flow cytometry. 19BBε WT CAR expressing primary T cells efficiently degranulated upon 3 hours of co-incubation with CD19^+^ Nalm6 cells (**Figures 6A, B**). However, primary T cells expressing the phospho-mimetic variants of 19BBε CARs (Y39E, Y50E, Y39/50E) failed to degranulate upon co-incubation with target cells (**Figures 6A, B**). Stimulation of the endogenous TCR with an anti-CD3 antibody was used as positive control and resulted in equal levels of degranulation in primary CAR T cells independent of the expressed 19BBε variants (**Figures 6A, B**). To account for the cytotoxicity of these constructs in a broader time window and to also consider alternative cytotoxic axes such as Fas/FasL, we performed a luciferase-based killing assay targeting CD19^+^ Nalm6 cells by measuring specific killing at several time points (**Figure 6C**). The killing of tumor cells by the primary T cells expressing the phospho-mimetic CARs (Y39E, Y50E, Y39/50E) was significantly reduced and delayed compared to CAR T cells expressing the 19BBε WT CAR. For instance, the 19BBε WT CAR T cells killed 100% of CD19-expressing target cells after 12 hours of co-incubation. In marked contrast, 19BBε Y39E, Y50E and Y39/50 CAR T cells killed only 20% of Nalm6 cells at this time point. Only after 52 hours of co-incubation, all CAR T cells expressing the three phospho-mimetic CARs killed 100% of the target cells (**Figure 6C**).

Next, we investigated the ability to secrete the pro-inflammatory cytokines IFNγ and TNF by primary CAR T cells expressing our novel 19BBε CARs. Primary CAR T cells were co-incubated with CD19^+^ Nalm6 cells for 24 hours, and cytokine secretion was assayed by ELISA. Primary human T cells expressing the 19BBε WT CAR efficiently secreted IFNγ and TNF upon CAR triggering (**Figures 6D, E**). In contrast, all three phospho-mimetic variants (Y39E, Y50E, Y39/50E) failed to transmit signals resulting in IFNγ or TNF production (**Figures 6D, E**). However, stimulation with PMA and ionomycin demonstrated equal potency to secrete IFNγ and TNF for all cells, independent of the expressed CAR construct indicating that all CAR T cells have the potential to be activated (**Figures 6D, E**). Altogether, primary T cells expressing 19BBε WT CARs are functional to eliminate tumor cells. However, mimicking irreversible phosphorylation of the 19BBε CAR impairs signal transduction, reducing and delaying cytotoxicity as well as preventing cytokine secretion.

## Discussion

The TCR-CD3 complex contains ten ITAMs and although these ITAMs share a conserved YxxL/I-X_6-8_-YxxL/I motif, the exact amino acid sequence of each of them is distinct, resulting in different binding affinities to signaling molecules. In contrast, other receptors of the immune system have significantly fewer ITAMs. For instance, the B cell receptor has only two ITAMs. The multitude of TCR ITAMs likely contributes to signal amplification, as suggested by murine models, which show that reducing the number of ITAMs below seven per TCR-CD3 complex leads to an autoimmune disorder, as a result of impaired TCR function during establishment of central tolerance in the thymus (22). Alternative evidence suggests that the different ITAMs may also have non-redundant functions. Namely, experiments in which all ITAMs of the TCR were exchanged by a given ITAM, support that ITAM diversity is relevant for signal transduction and T cell development even when the total number of ITAMs is conserved (30).

Upon TCR binding to its ligand (pMHC), ITAMs are phosphorylated by LCK (18, 19, 31), allowing the recruitment of SH2 domain-containing proteins. However, how LCK is recruited to the TCR-CD3 complex, to phosphorylate the ITAMs, is still not fully understood. Several models have been proposed to mechanistically explain this crucial step. For instance, the formation of a tri-molecular complex of the TCR with pMHC and the co-receptors CD4 or CD8, bringing co-receptor-associated LCK close to the TCR to initiate phosphorylation. However, most recent evidences support that the formation of this tri-molecular complex is a consequence of ITAM phosphorylation. In this scenario, the driving force of the tri-molecular complex is the co-receptor-bound LCK that interacts via its SH2 domain with the phosphorylated ITAMs (32–36). In another model, LCK randomly encounters TCRs by diffusion (37). Furthermore, ITAM phosphorylation is controlled by conformational changes within the TCR-CD3 complex regulating the accessibility of the ITAMs (9, 12, 38–40). Additionally, stabilization of the active TCR conformation exposes the PRS motif, which overlaps with the N-terminal half of the CD3ε ITAM, leading to the recruitment of NCK via its SH3.1 domain (6). We have recently identified the RK motif (RKxQRxxxY), which overlaps with the C-terminal half of the CD3ε ITAM (8). Upon ligand-binding to the TCR, the RK motif is exposed and recruits LCK by a non-canonical interaction that involves the SH3 domain of LCK and the un-phosphorylated CD3ε ITAM. Therefore, ITAMs might have functions prior to their phosphorylation and the evidence suggests that the CD3ε ITAM might function as a molecular switch, tightly regulating the CD3ε signalosome upon TCR engagement.

Herein, we aimed to investigate how phosphorylation of each of the tyrosines of the CD3ε ITAM impacts TCR signal initiation. Our working hypothesis was that protein interactions with the un-phosphorylated and mono-phosphorylated CD3ε ITAM constitute a crucial step to initiate TCR downstream signals. To this end, we embraced an approach to mimic irreversible phosphorylation of the CD3ε ITAM by exchanging its tyrosines to glutamic acid. Our results validate the suitability of this strategy. The tandem SH2 domains of ZAP70 have been reported to interact with the double-phosphorylated ITAM of CD3ε (15), while they hardly bind to mono-phosphorylated ITAMs (16). Interestingly, the ITAM of CD3ε has the lowest affinity to ZAP70 among the TCR ITAMs (41). As expected, ZAP70 bound better to the double-mutated CD3ε ITAM variant (Y39/50E), than to the other CD3ε variants or εWT. Un-phosphorylated CD3ε recruits NCK via its SH3.1 domain to the PRS motif. This interaction is further stabilized by binding of the NCK SH2 domain to the phosphorylated second tyrosine (Y50) of CD3ε (21), while phosphorylation of the first tyrosine of this ITAM (Y39) disrupts NCK binding to CD3ε (20). Indeed, we confirmed these results by showing that NCK bound to the un-phosphorylated CD3ε WT ITAM and this binding was enhanced by the Y50E mutation. Upon ligand-binding to the TCR, the RK motif is exposed and recruits LCK via its SH3 domain to the un-phosphorylated CD3ε ITAM. Molecular modeling predicted that phosphorylation of Y50 will prevent this interaction, and experimental evidence showed that LCK preferentially binds to non-phosphorylated CD3ε over double-phosphorylated CD3ε (8). Here, we have systematically assessed the role of the CD3ε ITAM tyrosines for LCK binding with our phospho-mimetic variants. In our pull-down experiments with CD3ε-GST fusion proteins, we demonstrated that LCK bound to the un-phosphorylated CD3ε WT and the phospho-mimetic CD3ε Y39E, while the variants Y50E and Y39/50E abolished LCK binding. Using our established TCR-LCK *in situ* PLA, we confirmed only residual binding (<30%) of LCK to the CD3ε ITAM when mono-phosphorylated at the C-terminal tyrosine (Y50E).

The impact of these variants in the context of the TCR-CD3 was clear but modest, since in the TCR-CD3 complex, eight additional ITAMs might compensate for these mutations (22). By expressing εWT or the phospho-mimetic CD3ε variants in a JK T cell line lacking endogenous CD3ε, we demonstrated that all CD3ε variants restored TCR assembly and expression at the T cell surface. Interestingly, the phospho-mimetic variants did not induce signaling in the absence of TCR engagement that would result in up-regulation of activation markers like CD69 or in IL-2 secretion, suggesting that the TCR-CD3 complex prevents spurious signals despite mimicking the phosphorylation of the CD3ε ITAM and the recruitment of ZAP70. Upon TCR engagement, the up-regulation of CD69, CD25 and 4-1BB was decreased by 10-40%, while IL-2 secretion by cells expressing the phospho-mimetic variants was 60% reduced compared to CD3ε WT. All phospho-mimetic CD3ε variants reduced ZAP70 phosphorylation by 30-50%, preventing optimal downstream signaling. These results highlight that reversible CD3ε phosphorylation is key to ensure optimal T cell activation. Previous reports have shown that phenylalanine substitution at Y39 abolished signal transduction, including the phosphorylation at the CD3ε C-terminal tyrosine, and ZAP70 association (42) pointing out the importance of the OH group of tyrosines for molecular interactions. Our data suggest different thresholds for the up-regulation of activation markers and the secretion of IL-2, indicating different degrees of compensation for the harmful effects of the phospho-mimetic CD3ε variant by the additional TCR ITAMs. In all, our results suggest that a reduction of 50% in signal transduction seems to be still enough to induce transcription of activation markers, but fails to induce cytokine secretion. Indeed, IL-2 expression upon TCR engagement is binary (all-or-none), while the upregulation of activation markers is graded and can be induced by suboptimal signals. Experimental evidences and mathematical modeling have suggested that TCR triggering results in digital NFAT activation, while NFκB activation is graded. Subsequently, NFAT translocates into the nucleus in an all-or-none fashion, transforming the strength of TCR-stimulation into the number of nuclei positive for activated NFAT and IL-2 transcription (43). Taken together, CD3ε phospho-mimetic variants are thus unable to efficiently transduce TCR-stimulation into downstream activation signals.

Due to the compensatory effect of the additional TCR-CD3 ITAMs, we decided to take advantage of CARs as minimal TCR constructs. In fact, very early studies, assessing the minimal requirements for T cell activation, set the path for engineering these synthetic receptors, demonstrating that the cytoplasmic tail of ζ, with its three ITAMs, is sufficient to drive T cell effector functions (44). Indeed, we and others have recently demonstrated that mechanistic principles discovered in the TCR are instrumental to improve the performance of CARs against cancer in pre-clinical models (8, 26). On the one hand, reduced tonic signaling and increased anti-tumor efficacy was achieved by introducing the RK motif of CD3ε into a second-generation FDA-approved 19BBζ CAR. Mechanistically, LCK recruitment to the CAR and its phosphorylation was better harmonized with CAR engagement (8). On the other hand, incorporating the CD3ε cytoplasmic tail in a second-generation FDA-approved CD28-based CAR enhanced persistence and decreased toxicity. Recruitment of CSK to the mono-phosphorylated CD3ε ITAM restrained excessive T cell activation and cytokine secretion (26). Still, our knowledge of the differences and similarities of how CARs and TCRs transmit activation signals is quite limited. When CARs and TCRs were stimulated with the same antigen concentrations, the TCR was more efficiently phosphorylated, and ZAP70 better recruited (45). Similarly, the adaptor protein LAT and PLCγ were only weakly phosphorylated by CAR stimulation when compared to TCR stimulation (46). Here, we have generated second-generation CARs containing only the cytoplasmic tail of CD3ε to avoid compensatory effects by additional ITAMs of the TCR-CD3 complex. Our CARs contain the signaling domain of 4-1BB, which activates the NFκB pathway. Indeed, we saw some degree of NFκB activation in the absence of ligand upon CAR expression independently of the CD3ε variant used. CAR Tonic signaling was reported to play a crucial role in regulating *in vivo* CAR T cell fitness and its anti-tumor function (47, 48). The phospho-mimetic CAR variants did not increased tonic signaling compared to the WT CAR, demonstrated by the lack of up-regulation of the activation marker CD69, CD25 and 4-1BB or the secretion of IL-2 in the basal conditions. Upon co-incubation with CD19-expressing tumor cells, the activation of NFκB and NFAT, the upregulation of CD69, CD25 and 4-1BB, and the secretion of IFNγ and TNF were severely impaired by the phospho-mimicking CAR variants compared to the WT CAR. The data provide evidence that for optimal T cell activation by CARs, dynamic CD3ε ITAM phosphorylation is essential. Likewise, CAR-induced T cell degranulation and tumor killing were strongly reduced in CAR T cells expressing the phospho-mimicking CAR variants. Substitution of Y39 to phenylalanine also diminished CAR T cell activation in a previous study (27), again supporting that dynamic CD3ε ITAM phosphorylation is key for the functioning of these CAR T cells. The mechanism how each phospho-mimetic variant reduced T cell activation differs. Y39E prevents NCK recruitment, Y50E prevents LCK recruitment, and Y39E/Y50E allows ZAP70 binding that cannot be optimally activated due to the absence of LCK. These results highlight the importance of the CD3ε signalosome to initiate T cell activation. The fact that the primary T cells expressing the phospho-mimetic CAR variants showed decreased secretion of pro-inflammatory cytokines and slower killing might be beneficial in some therapeutic settings. Therefore, we think that it is worthwhile to test these constructs in pre-clinical models that not only assess for anti-tumor effects, but in humanized models that can also assay CAR T cell-associated toxicities. The current notion is that excessive CAR T cell activation leads to T cell dysfunction or exhaustion, to shorter survival due to activation-induced apoptosis, and to harmful cytokine secretion inducing a life-threating cytokine release syndrome. Nevertheless, these studies are out of the scope of this work.

In all, our data support the notion that the CD3ϵ ITAM might has evolved to facilitate initiation of TCR signaling prior to its phosphorylation unravelling a phosphorylation-independent function of an ITAM. Thus, the CD3ε ITAM is a unique and multifunctional switch fine-tuning TCR downstream signaling and T cell activation.

## Data availability statement

The original contributions presented in the study are included in the article/**Supplementary Material**. Further inquiries can be directed to the corresponding author.

## Ethics statement

The studies involving humans were approved by Albert-Ludwigs-Universität Freiburg Ethik-Kommission. The studies were conducted in accordance with the local legislation and institutional requirements. The human samples used in this study were acquired from a by-product of routine care or industry. Written informed consent for participation was not required from the participants or the participants’ legal guardians/next of kin in accordance with the national legislation and institutional requirements. Ethical approval was not required for the studies on animals in accordance with the local legislation and institutional requirements because only commercially available established cell lines were used.

## Author contributions

SM: Conceptualization, Formal analysis, Funding acquisition, Investigation, Project administration, Resources, Supervision, Writing – original draft, Writing – review & editing. NMW: Data curation, Formal analysis, Methodology, Validation, Visualization, Writing – review & editing. SMB: Data curation, Formal analysis, Methodology, Validation, Writing – review & editing. SH: Data curation, Writing – review & editing. WWS: Funding acquisition, Supervision, Writing – review & editing. FAH: Conceptualization, Investigation, Supervision, Writing – original draft.

## Supplementary material

Supplementary Figure 1 Generation of JK T cells expressing phospho-mimetic CD3ε variants. **(A)** Flow cytometry analysis of zsGreen expression and TCR (CD3ε) surface levels in JK εKO cells lentivirally transduced with the indicated hCD3ε constructs. Untransduced JK εKO and JK cells (UTD) were used as negative controls. **(B)** Percentage of zsGreen^+^ cells. **(C)** Mean fluorescence intensity (MFI) of zs Green in zsGreen^+^ cells. **(D)** Percentage of CD3ε^+^ cells. **(E)** MFI of CD3ε in CD3ε^+^ cells. **(F)** Percentage of CD28^+^ cells. **(G)** MFI of CD28 in CD28^+^ cells. Each dot represents one independent experiment. Mean values ± SD are indicated. One-way ANOVA test was performed after Shapiro-Wilk test for normality. * P < 0.05.

Supplementary Figure 2 Generation of JK NFκB and NFAT εKO reporter cell lines expressing phospho-mimetic CD3ε variants. **(A)** Representative dot plots showing TCR (CD3ε) expression in JK NFκB and NFAT reporter cells before and after CRISPR/Cas9 KO of CD3ε. Unstained JK reporter cells were used as negative control. **(B)** Percentage of CD3ε^+^ cells and **(C)** CD3ε expression after CRISPR/Cas9 KO of CD3ε. **(D)** Representative dot plots showing BFP levels and TCR (CD3ε) surface levels in JK NFκB εKO reporter cells lentivirally transduced with the indicated hCD3ε constructs. **(E)** Percentage of BFP^+^ cells. **(F)** MFI of BFP in BFP^+^ cells. **(G)** Percentage of CD3ε^+^ cells. **(H)** MFI of CD3ε in CD3ε^+^ cells. **(I)** Representative dot plots showing BFP expression and TCR (CD3ε) surface levels in JK NFAT εKO reporter cells lentivirally transduced with the indicated hCD3ε constructs. **(J)** Percentage of BFP^+^ cells. **(K)** MFI of BFP in BFP^+^ cells. **(L)** Percentage of CD3ε^+^ cells. **(M)** MFI of CD3ε in CD3ε^+^ cells. One-way ANOVA with Dunnett’s multiple comparisons test was performed after Shapiro-Wilk test for normality. Mean values ± SD are indicated. Each dot represents one independent experiment. **P < 0.01, ****P < 0.0001.

Supplementary Figure 3 LCK phosphorylation is unaffected by the expression of phospho-mimetic CD3ε variants. JK εKO cells expressing the respective hCD3ε variants were left unstimulated or stimulated with 5 μg/ml anti-CD3 antibody for 5 min. Total cell lysates were subjected to immunoblotting with anti-pLCK (Y394 and Y505), anti-LCK and anti-GAPDH antibodies. JK εKO cells were used as negative control. The quantification of 6 independent experiments normalized to the stimulated WT is shown. Two-way ANOVA with Dunnett’s multiple comparisons test was performed after Shapiro-Wilk test for normality. Mean values ± SD are indicated. Each dot represents one independent experiment.

Supplementary Figure 4 Generation of JK T cells expressing phospho-mimetic 19BBε CAR variants. Flow cytometry analysis in JK cells lentivirally transduced with the indicated 19BBε CAR construct. Untransduced JK cells (UTD) were used as negative control. **(A)** Representative dot plots showing GFP levels and CAR surface levels. **(B)** Percentage of GFP^+^ cells. **(C)** Mean fluorescence intensity (MFI) of GFP in GFP^+^ cells. **(D)** Percentage of CAR^+^ cells. **(E)** MFI of CAR in CAR^+^ cells. **(F)** Percentage of CD28^+^ cells. **(G)** MFI of CD28 in CD28^+^ cells. Each dot represents one independent experiment. One-way ANOVA test was performed after Shapiro-Wilk test for normality. Mean values ± SD are indicated. Each dot represents one independent experiment. *P < 0.05  

Supplementary Figure 5 Generation of JK NFκB and NFAT εKO reporter cell lines expressing phospho-mimetic 19BBε CARs. **(A)** Representative dot plots showing BFP levels and CAR surface levels in JK NFκB εKO reporter cells lentivirally transduced with the indicated 19BBε CAR constructs. **(B)** Percentage of BFP^+^ cells. **(C)** MFI of BFP in BFP^+^ cells. **(D)** Percentage of CAR^+^ cells. **(E)** MFI of CAR in CAR^+^ cells. **(F)** Representative dot plots showing BFP levels and CAR surface levels in JK NFAT εKO reporter cells lentivirally transduced with the indicated 19BBε CAR constructs. **(G)** Percentage of BFP^+^ cells. **(H)** MFI of BFP in BFP^+^ cells. **(I)** Percentage of CAR^+^ cells. **(J)** MFI of CAR in CAR^+^ cells. One-way ANOVA with Dunnett’s multiple comparisons test was performed after Shapiro-Wilk test for normality. Mean values ± SD are indicated. Each dot represents one independent experiment. *P < 0.5, **P < 0.01, ****P < 0.0001.

Supplementary Figure 6 Generation of primary human T cells expressing phospho-mimetic 19BBε CAR variants. **(A)** Representative dot plots showing GFP levels and CAR surface levels in primary human T cells lentivirally transduced with the indicated 19BBε CAR constructs. Untransduced (UTD) as well as Mock transduced primary T cells were used as negative controls. **(B)** Percentage of GFP^+^ cells. **(C)** MFI of GFP in GFP^+^ cells. **(D)** Percentage of CAR^+^ cells. **(E)** MFI of CAR in CAR^+^ cells. Each dot represents one healthy donor. One-way ANOVA test was performed after Shapiro-Wilk test for normality. Mean values ± SD are indicated. *P < 0.5, **P < 0.01, ****P < 0.0001.
