# Supplementary figures and images for: Phospho-mimetic CD3ε variants prevent TCR and CAR signaling
Source: Front Immunol. 2024 May 8;15:1392933. doi: 10.3389/fimmu.2024.1392933 (PMC11109380; doi:10.3389/fimmu.2024.1392933)

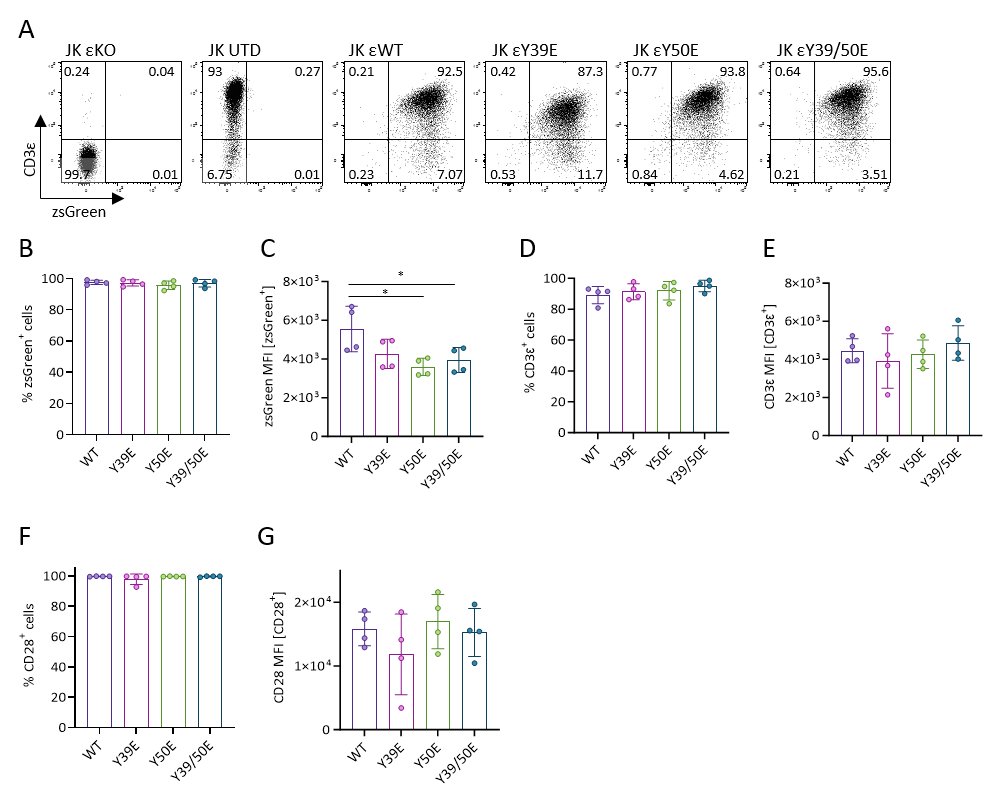

Supplement: Supplementary Figure 1 — Generation of JK T cells expressing phospho-mimetic CD3ε variants. (A) Flow cytometry analysis of zsGreen expression and TCR (CD3ε) surface levels in JK εKO cells lentivirally transduced with the indicated hCD3ε constructs. Untransduced JK εKO and JK cells (UTD) were used as negative controls. (B) Percentage of zsGreen+ cells. (C) Mean fluorescence intensity (MFI) of zs Green in zsGreen+ cells. (D) Percentage of CD3ε+ cells. (E) MFI of CD3ε in CD3ε+ cells. (F) Percentage of CD28+ cells. (G) MFI of CD28 in CD28+ cells. Each dot represents one independent experiment. Mean values ± SD are indicated. One-way ANOVA test was performed after Shapiro-Wilk test for normality. * P < 0.05. [file Image_1.tif]

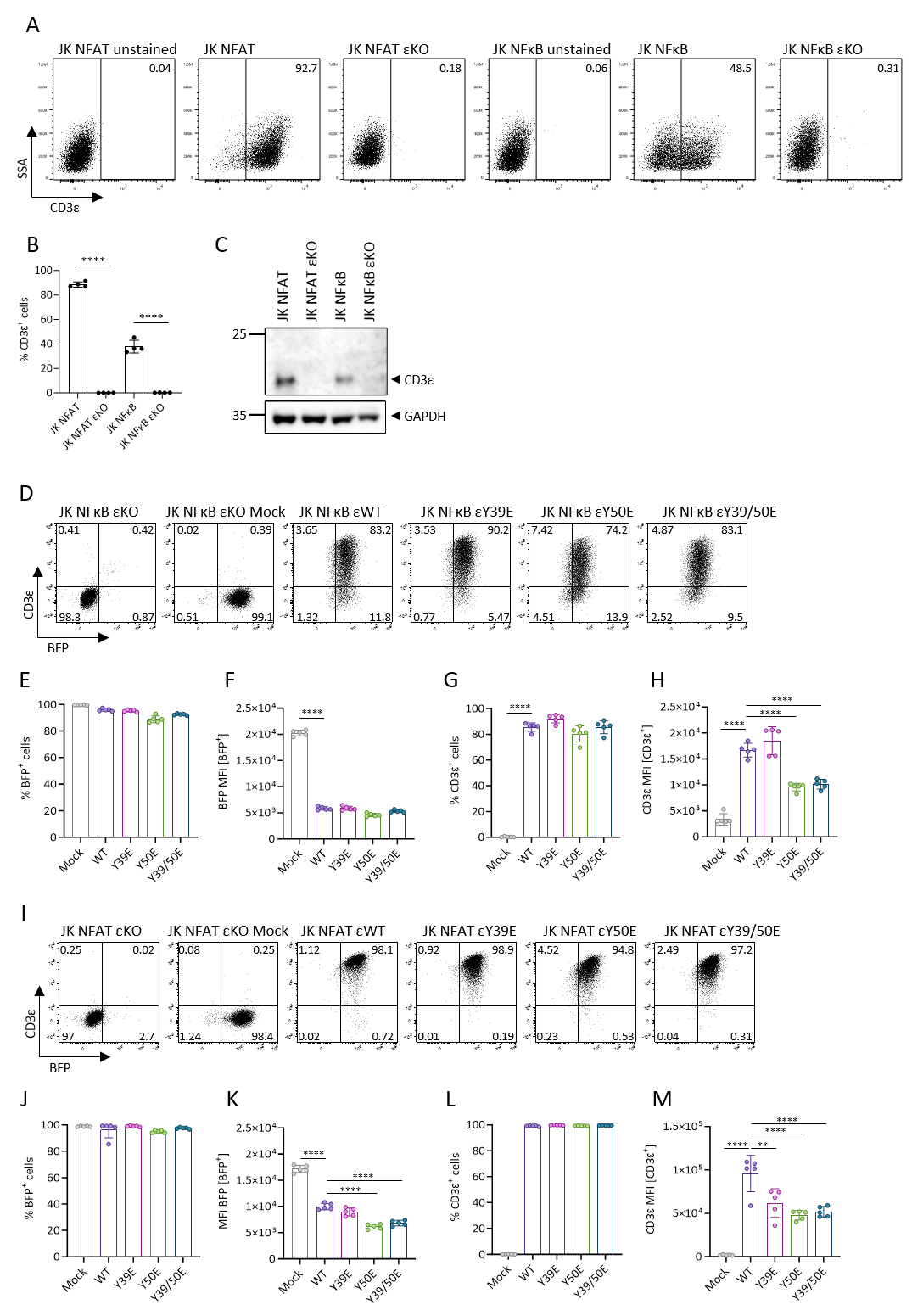

Supplement: Supplementary Figure 2 — Generation of JK NFκB and NFAT εKO reporter cell lines expressing phospho-mimetic CD3ε variants. (A) Representative dot plots showing TCR (CD3ε) expression in JK NFκB and NFAT reporter cells before and after CRISPR/Cas9 KO of CD3ε. Unstained JK reporter cells were used as negative control. (B) Percentage of CD3ε+ cells and (C) CD3ε expression after CRISPR/Cas9 KO of CD3ε. (D) Representative dot plots showing BFP levels and TCR (CD3ε) surface levels in JK NFκB εKO reporter cells lentivirally transduced with the indicated hCD3ε constructs. (E) Percentage of BFP+ cells. (F) MFI of BFP in BFP+ cells. (G) Percentage of CD3ε+ cells. (H) MFI of CD3ε in CD3ε+ cells. (I) Representative dot plots showing BFP expression and TCR (CD3ε) surface levels in JK NFAT εKO reporter cells lentivirally transduced with the indicated hCD3ε constructs. (J) Percentage of BFP+ cells. (K) MFI of BFP in BFP+ cells. (L) Percentage of CD3ε+ cells. (M) MFI of CD3ε in CD3ε+ cells. One-way ANOVA with Dunnett’s multiple comparisons test was performed after Shapiro-Wilk test for normality. Mean values ± SD are indicated. Each dot represents one independent experiment. **P < 0.01, ****P < 0.0001. [file Image_2.tif]

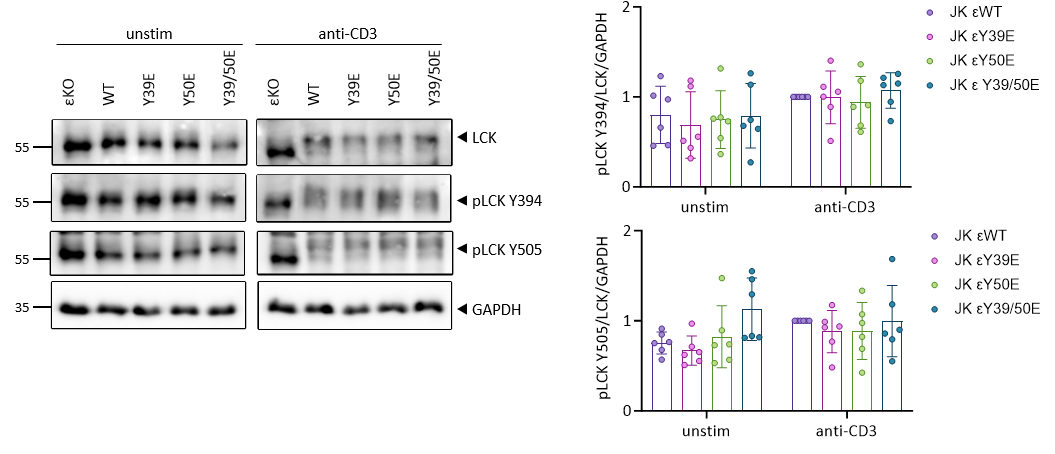

Supplement: Supplementary Figure 3 — LCK phosphorylation is unaffected by the expression of phospho-mimetic CD3ε variants. JK εKO cells expressing the respective hCD3ε variants were left unstimulated or stimulated with 5 μg/ml anti-CD3 antibody for 5 min. Total cell lysates were subjected to immunoblotting with anti-pLCK (Y394 and Y505), anti-LCK and anti-GAPDH antibodies. JK εKO cells were used as negative control. The quantification of 6 independent experiments normalized to the stimulated WT is shown. Two-way ANOVA with Dunnett’s multiple comparisons test was performed after Shapiro-Wilk test for normality. Mean values ± SD are indicated. Each dot represents one independent experiment. [file Image_3.tif]

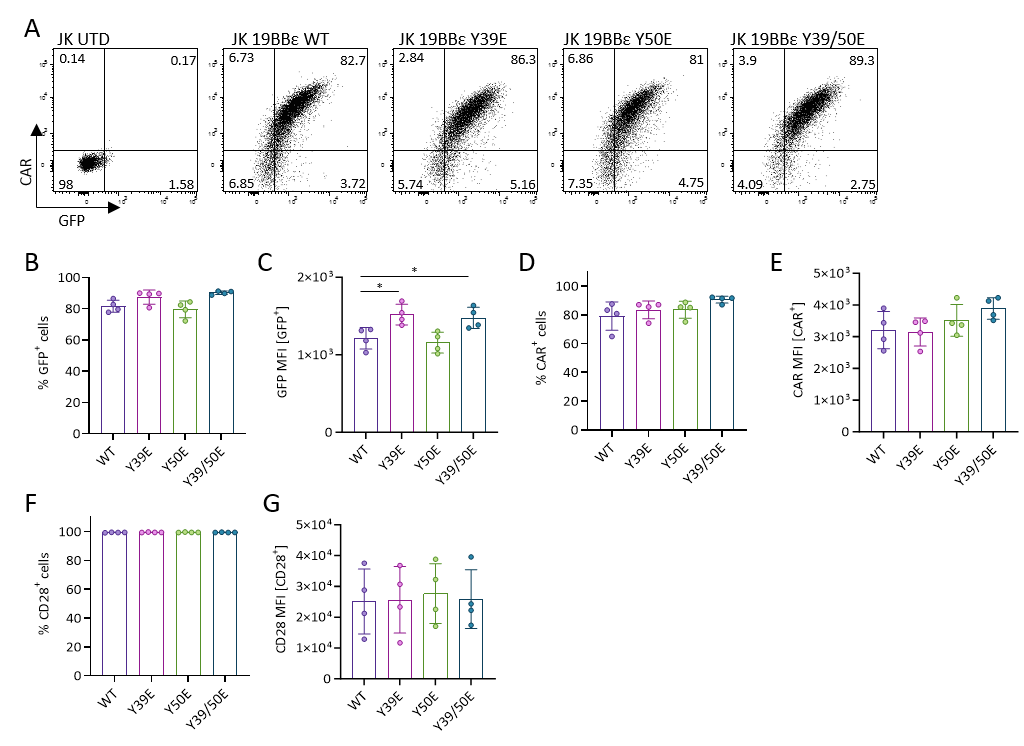

Supplement: Supplementary Figure 4 — Generation of JK T cells expressing phospho-mimetic 19BBε CAR variants. Flow cytometry analysis in JK cells lentivirally transduced with the indicated 19BBε CAR construct. Untransduced JK cells (UTD) were used as negative control. (A) Representative dot plots showing GFP levels and CAR surface levels. (B) Percentage of GFP+ cells. (C) Mean fluorescence intensity (MFI) of GFP in GFP+ cells. (D) Percentage of CAR+ cells. (E) MFI of CAR in CAR+ cells. (F) Percentage of CD28+ cells. (G) MFI of CD28 in CD28+ cells. Each dot represents one independent experiment. One-way ANOVA test was performed after Shapiro-Wilk test for normality. Mean values ± SD are indicated. Each dot represents one independent experiment. *P < 0.05 [file Image_4.tif]

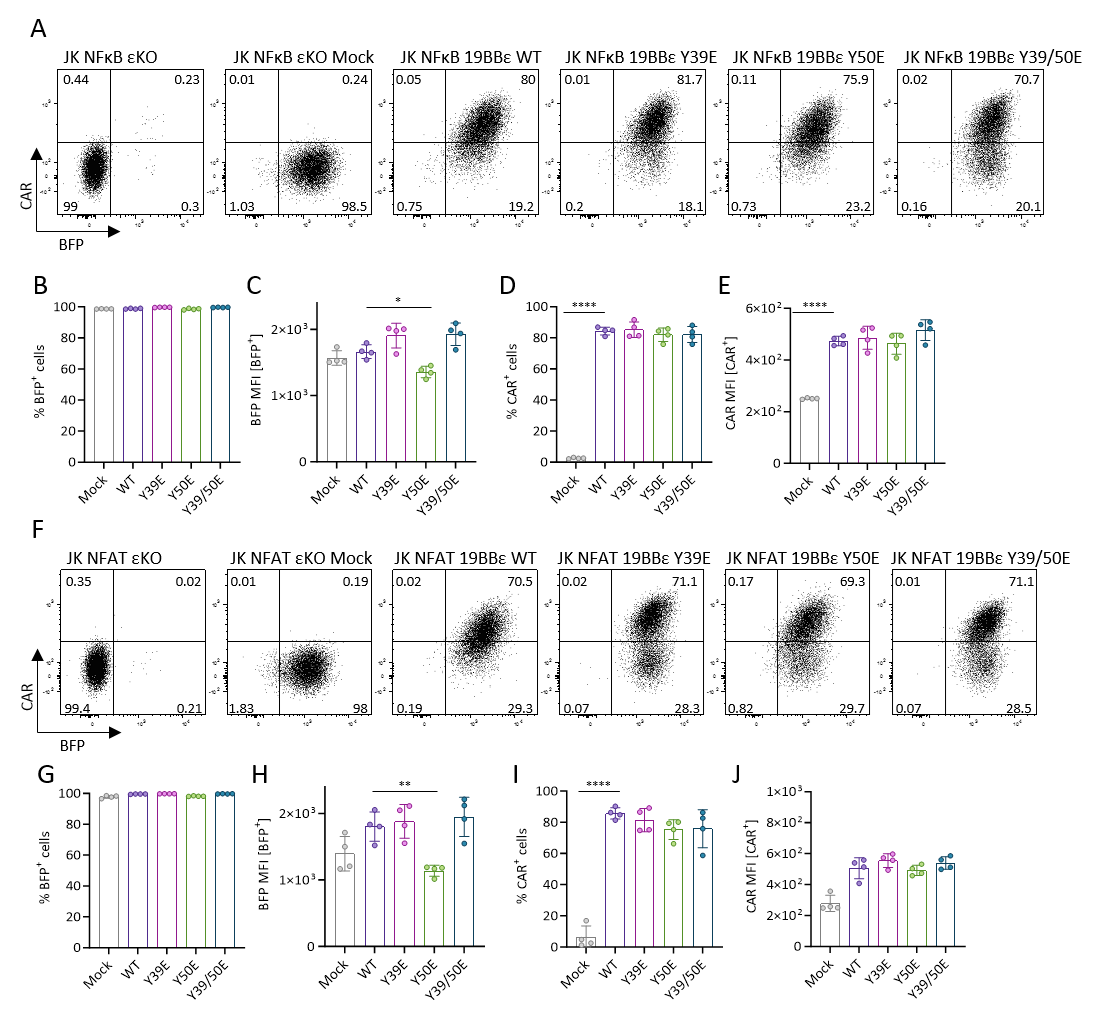

Supplement: Supplementary Figure 5 — Generation of JK NFκB and NFAT εKO reporter cell lines expressing phospho-mimetic 19BBε CARs. (A) Representative dot plots showing BFP levels and CAR surface levels in JK NFκB εKO reporter cells lentivirally transduced with the indicated 19BBε CAR constructs. (B) Percentage of BFP+ cells. (C) MFI of BFP in BFP+ cells. (D) Percentage of CAR+ cells. (E) MFI of CAR in CAR+ cells. (F) Representative dot plots showing BFP levels and CAR surface levels in JK NFAT εKO reporter cells lentivirally transduced with the indicated 19BBε CAR constructs. (G) Percentage of BFP+ cells. (H) MFI of BFP in BFP+ cells. (I) Percentage of CAR+ cells. (J) MFI of CAR in CAR+ cells. One-way ANOVA with Dunnett’s multiple comparisons test was performed after Shapiro-Wilk test for normality. Mean values ± SD are indicated. Each dot represents one independent experiment. *P < 0.5, **P < 0.01, ****P < 0.0001. [file Image_5.tif]

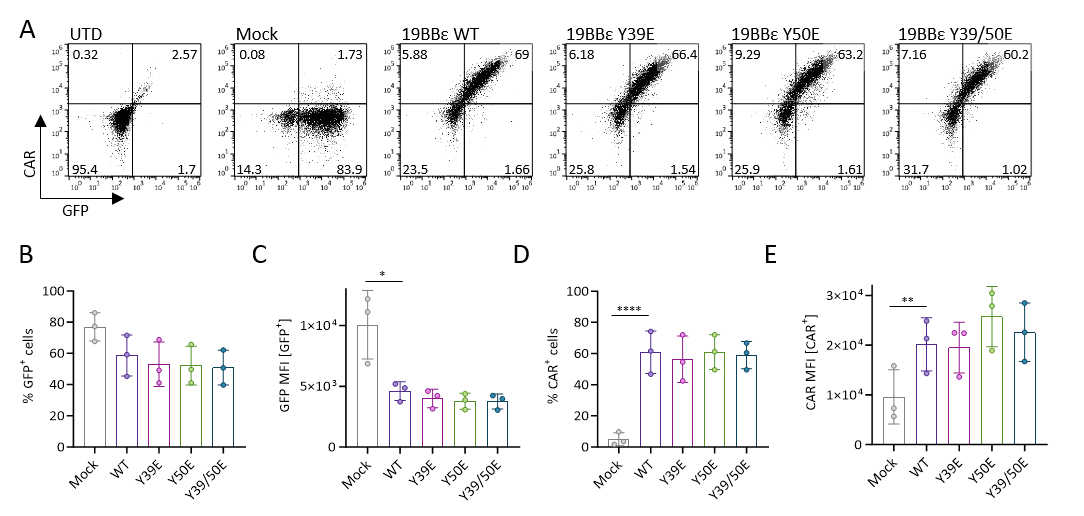

Supplement: Supplementary Figure 6 — Generation of primary human T cells expressing phospho-mimetic 19BBε CAR variants. (A) Representative dot plots showing GFP levels and CAR surface levels in primary human T cells lentivirally transduced with the indicated 19BBε CAR constructs. Untransduced (UTD) as well as Mock transduced primary T cells were used as negative controls. (B) Percentage of GFP+ cells. (C) MFI of GFP in GFP+ cells. (D) Percentage of CAR+ cells. (E) MFI of CAR in CAR+ cells. Each dot represents one healthy donor. One-way ANOVA test was performed after Shapiro-Wilk test for normality. Mean values ± SD are indicated. *P < 0.5, **P < 0.01, ****P < 0.0001. [file Image_6.tif]
